# Almost a spider: a 305-million-year-old fossil arachnid and spider origins

**DOI:** 10.1098/rspb.2016.0125

**Published:** 2016-03-30

**Authors:** Russell J. Garwood, Jason A. Dunlop, Paul A. Selden, Alan R. T. Spencer, Robert C. Atwood, Nghia T. Vo, Michael Drakopoulos

**Affiliations:** 1 School of Earth, Atmospheric and Environmental Sciences, University of Manchester, Manchester M13 9PL, UK; 2 Museum für Naturkunde, Leibniz Institute for Research on Evolution and Biodiversity, Invalidenstraße 43, 10115 Berlin, Germany; 3 Department of Geology, University of Kansas, Lindley Hall, 1475 Jayhawk Boulevard, Lawrence, KS 66045, USA; 4 The Natural History Museum, London SW7 5BD, UK; 5 Department of Earth Sciences and Engineering, Imperial College London, London, UK; 6 Diamond Light Source, The Harwell Science and Innovation Campus, Didcot, Oxfordshire OX11 0DE, UK

**Keywords:** Araneae, Pantetrapulmonata, Arachnida, Carboniferous, Montceau-les-Mines

## Abstract

Spiders are an important animal group, with a long history. Details of their origins remain limited, with little knowledge of their stem group, and no insights into the sequence of character acquisition during spider evolution. We describe a new fossil arachnid, *Idmonarachne brasieri* gen. et sp. nov. from the Late Carboniferous (Stephanian, *ca* 305–299 Ma) of Montceau-les-Mines, France. It is three-dimensionally preserved within a siderite concretion, allowing both laboratory- and synchrotron-based phase-contrast computed tomography reconstruction. The latter is a first for siderite-hosted fossils and has allowed us to investigate fine anatomical details. Although distinctly spider-like in habitus, this remarkable fossil lacks a key diagnostic character of Araneae: spinnerets on the underside of the opisthosoma. It also lacks a flagelliform telson found in the recently recognized, spider-related, Devonian–Permian Uraraneida. Cladistic analysis resolves our new fossil as sister group to the spiders: the spider stem-group comprises the uraraneids and *I. brasieri*. While we are unable to demonstrate the presence of spigots in this fossil, the recovered phylogeny suggests the earliest character to evolve on the spider stem-group is the secretion of silk. This would have been followed by the loss of a flagelliform telson, and then the ability to spin silk using spinnerets. This last innovation defines the true spiders, significantly post-dates the origins of silk, and may be a key to the group's success. The Montceau-les-Mines locality has previously yielded a mesothele spider (with spinnerets). Evidently, Late Palaeozoic spiders lived alongside Palaeozoic arachnid grades which approached the spider condition, but did not express the full suite of crown-group autapomorphies.

## 1. Introduction

Spiders (Arachnida: Araneae) are a diverse and successful arthropod clade, which can be traced back *ca* 315 Ma to the Late Carboniferous [1]. Many uncertainties surround spider origins, but the clade is probably closely related to the recently recognized Devonian–Permian Uraraneida (known from *ca* 385 Ma to *ca* 275 Ma; [2])—arachnids that resembled spiders, but retained a flagelliform telson. While uraraneids had silk–producing spigots, they lacked spinnerets (abdominal appendages that allow increased control over silk production). One of the oldest reported spiders was found in the Late Carboniferous (*ca* 305 Ma) deposits of Montceau-les-Mines in France. This important Konservat-Lagerstätte has yielded scorpions [3], harvestmen [4,5] and members of the extinct order Trigonotarbida [6], in addition to other invertebrates [7,8], vertebrates [9,10] and plants [11]. The spider discovered at the site is explicitly referable to the earliest-branching spider suborder Mesothelae [12]. Because Montceau fossils are generally preserved three-dimensionally in siderite (ironstone) concretions, fine anatomical details can be recovered that allow precise systematic placement. The study of such fossils is enhanced by computed tomography (CT; [2,13,14]), which allows the void within the nodule to be mapped, creating a virtual fossil from the tomographic dataset (see Material and methods; [15]). Here, with the aid of laboratory and synchrotron CT, we report a new arachnid species from Montceau-les-Mines. We place the new species using a cladistic analysis, and discuss its impact on our understanding of spider origins.

## 2. Results

### (a) Morphological interpretation

**Figure 1.**
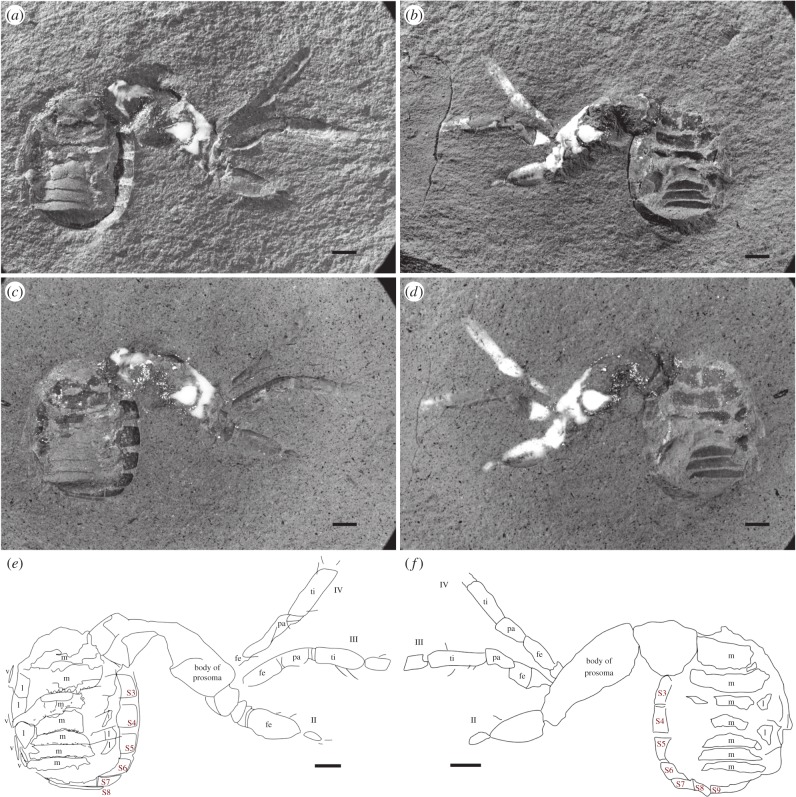
*Idmonarachne brasieri* gen. et sp. nov., Late Carboniferous of Montceau-les-Mines, France; part (*a*,*c*,*e*) and counterpart (*b*,*d*,*f*). (*a*,*b*) Dry in low-angle light, showing dorsal opisthosomal segmentation and surface relief; (*c*,*d*) under alcohol, showing leg setae and ventral segmentation more clearly; (*e*,*f*) morphological interpretation of fossil. All scale bars 1 mm. II, III, IV, second, third and fourth legs; fe, femur; l, lateral part of tergite; m, median part of tergite; pa, patella; S3–S9, ventral plates 3–9; ti, tibia.

**Figure 2.**
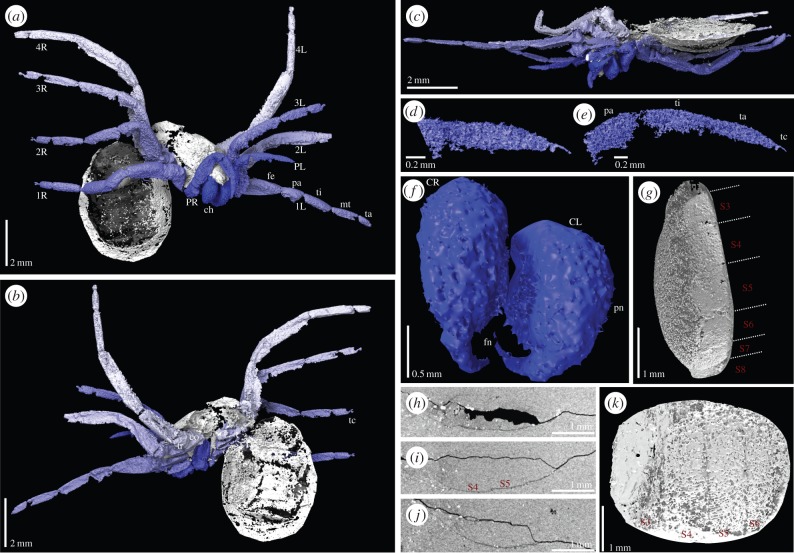
Digital visualization of *Idmonarachne brasieri* gen. et sp. nov. based on laboratory and synchrotron scans of the fossil. (*a*–*c*) Laboratory-based scans. (*a*) Prosoma in anterior view, and ventral opisthosoma of the specimen, with chelicerae tucked between pedipalps, ventral to the clypeus. (*b*) Dorsal opisthosoma, and prosoma in posterior view, showing some opisthosomal segmentation. (*c*) Ventral view of prosoma, leg coxae and cheliceral termination apparent. (*d*–*k*) Synchrotron scans. (*d*–*e*) Tips of pedipalps showing claw and onychium. (*f*) Isolated chelicerae in detail, comprising paturon and fang. (*g*) Lateral view of ventral opisthosoma, ventral plates numbered as described in the text—fourth and fifth lacking spinnerets. (*h*)–(*j*) Computed slice images showing the opisthosoma in cross section, posterior right, ventral bottom. Sternal plates are preserved as thin, but continuous pieces of cuticle. (*k*) Ventral view of opisthosoma, ventral plates numbered—fourth and fifth lacking spinnerets. 1L–4L, first to fourth left leg; 1R–4R, first to fourth right leg; ch, chelicerae; CL, left chelicera; CR, right chelicera; co, coxa; fe, femur; fn, fang; pa, patella; PL, left pedipalp; pn, paturon; PR, right pedipalp; S3–S9, ventral plates 3–9; tc, terminal claw; ti, tibia; tr, trochanter.

The specimen is nearly complete, but the opisthosoma is folded at almost right angles to the prosoma (figures 1 and 2*a*; electronic supplementary material, SI File 01), which is slightly laterally compressed. Details of eyes are neither visible in the hand specimen nor resolved in the CT scans. Fine details of the legs, especially the terminal portions, are lacking because these are truncated due to field of view limitations in the synchrotron scan. Nevertheless, the prosomal appendages are distinctly spider-like, and dissimilar to those of the common Carboniferous trigonotarbids (e.g. [16]). The chelicerae are large and robust, approaching the aranean plagiognath condition (*sensu* [17]). The pedipalps are considerably shorter than the legs (those of trigonotarbids are shorter but less so). Both the shapes and differentiation of the leg and pedipalp podomeres are much more distinct and spider-like than in other arachnids. The femora are thickened proximo-ventrally, tapering slightly distally towards the very short patella, which is subtriangular in side profile, and show a wide femur–patella joint with a dorsal hinge (in trigonotarbids the podomeres are more even in thickness along their length). The leg femora, tibiae and metatarsi are relatively long compared with the shorter tarsus (which in trigonotarbids is equal to or longer than the metatarsus: i.e. essentially less differentiated). The NHM scan reveals two tarsal claws, similar in position to the paired main claws observed in spiders, but the resolution is insufficient to demonstrate the presence or the absence of a middle claw (there is the suggestion of one on right leg II).

### (b) Absences and uncertainties

The exact nature of the opisthosomal tergites is somewhat obscure because the specimen has been subject to a degree of post-mortem distortion. The lateral dorsal surfaces are poorly preserved, especially towards the posterior. The median portions of the tergites curve downwards laterally, as in trigonotarbids, beyond which are remnants of a series of lateral, inflected areas which could be interpreted as lateral plates, especially on the left-hand anterior opisthosoma of the counterpart (figure 1). In order to provide protection with rigid plates yet allow flexure for expansion of the opisthosoma (e.g. following a meal, or in gravid females), a row of lateral plates is necessary, as seen in trigonotarbids and ricinuleids, for example, and consistent with the interpretation of dorsal tergites divided into median and lateral regions. Should there be no lateral plates, soft membrane must fill the gap between the lateral edges of better-preserved median tergal regions, where they curve downwards, and the ventral plates; this seems unlikely given the protective function of the sclerotized plates. It is the collapse of membranes between the median and lateral plates that is responsible for the post-mortem distortion of the dorsal surface. There is no such effect on the ventral surface, which is undistorted and continuous (figure 2*h*–*j*)—a fact we expand upon below. As a result of the distortion to the dorsal opisthosoma, coupled with the path of the crack along which the nodule was split, it is unlikely that the tergites visible in the fossil reflect the number in life. Were this the case, the total length of visible tergites would necessitate large expanses of soft membranes between the plates, reducing their protective function and contra the pattern seen in trigonotarbids. Additionally, no pantetrapulmonates with tergites have as few as seven. Based on this reasoning, and using trigonotarbids as a comparison [18], the idealized reconstruction presented herein possesses nine tergites and a telson.

Neither the hand specimen nor the CT scans show any trace of spinnerets, a telson, spiracles or an anal tubercle. Three of these are reflected by characters in our cladistic analysis, and thus have the potential to impact on the placement of this fossil, while all are important to consider when assessing the sequence of character acquisition on the spider stem. As such, we explain here our rationale when considering whether these are genuine, or taphonomic absences. In contrast to spiders (including mesotheles), this fossil has ventral plates, which are clearly visible in the part and counterpart of the hand specimen (figure 1, S3–S9; electronic supplementary material), and the CT-based reconstruction (figure 2, S3–S9; electronic supplementary material). Owing to variability in the number of visible opisthosomal segments among arachnids, the number scheme we present is based on counting from the anterior. We assume that the first opisthosomal ventral plate is incorporated into a pedicel, and that the first, short ventral plate, visible in the CT scan is that of opisthosomal segment two (figure 2*g*; or anterior operculum assuming homology of ventral plates with other pantetrapulmonates). Hence, the anteriormost ventral plate visible in the hand specimen would be opisthosomal ventral plate three (figure 1*e*,*f*; posterior operculum). Spiders universally possess spinnerets on opisthosomal segments four and five [2]. In any realistic numbering scheme for the visible ventral plates, four and five are visible in their entirety in the CT scan (figure 2*g*), and lack spinnerets. Spinnerets are clearly present in *Palaeothele montceauensis*, the earliest known mesothele spider, also from Moncteau-les-Mines [12]. These are preserved as structures between 0.2 and 0.6 mm in diameter at their base. Even if spinnerets an order of magnitude smaller than those of *P. montceauensis* were present in this fossil, they would remain comfortably within the resolving power of this 5 µm voxel scan. The ventral surface of the fossil is preserved as a thin layer of cuticle. As such, when thresholded to create a digital visualization, the resulting surface appears patchy: this is an artefact of the reconstruction process. Figure 2*h*–*j*, and the accompanying video (electronic supplementary material, SI File 02; via Dryad, http://dx.doi.org/10.5061/dryad.v089t/2), shows slice images from the scans, demonstrating that the ventral plates are continuous in the underlying data. If spinnerets were lost prior to fossilization we would expect disruption of these plates, surface topography associated with the missing structures, and a hole where they once attached. None are present, in either the three-dimensional reconstruction, or any slices. For this reason, we have coded—and consider—spinnerets genuinely absent in this fossil. Similarly, uraraneids possess a terminal flagelliform telson in excess of 100 µm in width, and several millimetres in length, which would be resolved here. If this flagellum were absent due to taphonomic loss, the model would possess the associated narrowing of the opisthosoma posteriorly, and a ring-like posterior segment. Both are lacking in this fossil, and thus, we consider the flagelliform telson genuinely absent. Spider spiracles are relatively small structures; those of the tarantula, while 0.6 mm long, are between 15 and 40 µm wide [19]. Assuming linear scaling with body length, those of this fossil would be roughly the width of a single voxel, and thus impossible to discern with any certainty in our highest resolution scans. We note that spiracles are not visible in any other Coal Measures arachnids reconstructed in this manner from this site [4,5] or elsewhere [20,21], even when visible in the hand specimen [22]. Furthermore, an apt comparison for this taxon would be to tetrapulmonate species that possess ventral plates. In such groups, such as amblypygids [23] and uropygids [24], the spiracles are obscured by the associated operculum, and thus not externally visible in extant, or fossil, specimens. These factors lead us to code these as an unknown, as opposed to a true absence, supported by the fact that spiracles are present in all extant tetrapulmonates. Similarly, arachnids have an anal opening, which is typically found on a tubercle in the pantetrapulmonates: it would be expected here, and has been resolved in tomographic reconstructions of trigonotarbid arachnids [13,25] and haptopods [26]. Our failure to resolve it is likely to stem from a combination of the distortion of the soft dorsal membrane, and the crack along which the nodule was originally split (figure 2*h*–*j*). This obscures details at the posteriormost opisthosomal margin, where the anal tubercle would most likely be found. While there is no character in our cladistic analysis to reflect the absence or the presence of an anal tubercle, we consider this to also be uncertain.

### (c) Systematic palaeontology

#### (i) Arachnida

Order uncertain

*Idmonarachne brasieri* gen. et sp. nov.

#### (ii) Etymology

Genus after wool-dyer *Idmon*, the father of *Arachne* in Greco-Roman mythology, to reflect the phylogenetic position of this genus as a close relative to the spiders. Species named in memory of the late Prof. Martin Brasier, of the University of Oxford, in recognition of his broad contributions to the study of ancient life.

#### (iii) Holotype, locality and age

MNHN-SOT MNHN.F.SOT110002. From the Montceau-les-Mines Lagerstätte (Massif Central, France), Assise de Montceau, Carboniferous, late Stephanian (=Gzhelian).

#### (iv) Diagnosis

Arachnid with clasp-knife chelicerae, showing an anteriorly projecting basal element, and with a bite oblique to the sagittal plane. Legs and pedipalps spider-like in form, distinctly shaped podomeres and joints as follows: pedipalp distinctly shorter than legs and tarsus not subdivided into metatarsus and tarsus; legs with femur slightly expanded proximo-ventrally; short, subtriangular patella with wide femur–patella joint; relatively elongate femur, tibia and metatarsus; and shorter tarsus with at least paired claws. Opisthosoma with dorsal tergites divided into median and lateral fields, and undivided ventral plates. Lacking a flagellum (cf. Uraraneida), and lacking spinnerets (cf. Araneae).

#### (v) Description

Total body length *ca* 10.4 mm (figures 1 and 2*a*; electronic supplementary material, SI File 01). Prosomal dorsal shield (carapace) preserved portion 4.7 mm long. Leg coxae surround a ventral plate-like sternum; length 1.5 mm, width 0.9 mm.

Chelicera of clasp-knife type (figure 2*f*), consisting of anteriorly directed basal paturon, length 1.0 mm, and distal fang, 0.7 mm long. Chelicera projects forwards and downwards, slightly splayed when viewed from above or below (figure 2*c*). Pedipalp pediform (figure 2*b*), slightly shorter than legs, total length 3.8 mm. Podomere lengths: femur 1.2 mm, patella 0.8 mm, tibia 1.0 mm, tarsus 0.8 mm. Pedipalp tarsus tapers somewhat to a point; single dorsal claw at pedipalp tip, situated on onychium (figure 2*d*,*e*). Legs pediform and fairly homogeneous. Leg formula (longest to shortest): IV, III, II, I. Podomere lengths of leg I (6.3 mm): femur 2.1 mm, patella 0.9 mm, tibia 1.6 mm, metatarsus 1.1 mm, tarsus 0.8 mm excluding terminal claw. Podomere lengths of leg II (6.4 mm): femur 1.7 mm, patella 1.0 mm, tibia 1.6 mm, metatarsus 1.4 mm, tarsus 0.7 mm excluding terminal claw. Podomere lengths of leg III (7.0 mm): femur 1.6 mm, patella 0.9 mm, tibia 1.8 mm, metatarsus 1.5 mm, tarsus 0.8 mm excluding terminal claw. Podomere lengths of leg IV (8.5 mm): femur 2.2 mm, patella 1.1 mm, tibia 2.3 mm, metatarsus 2.1 mm, tarsus 0.8 mm. All tarsi bear terminal claws, situated on onychium (figure 2*a*; electronic supplementary material, SI File 01). At least femora and tibiae bear curved macrosetae (figure 1*c*,*e*).

Opisthosoma suboval in outline; length 5.7 mm, maximum width 4.3 mm. At least seven tergites, all with straight posterior margin; anterior two significantly longer than those following. Dorsal tergites divided into medial and lateral plates; medial section convex-upwards; lateral section apparently flat and directed dorsolaterally, producing a wide W-shaped profile to the dorsal surface. Posteriormost tergite directed at a sharp angle downwards posteriorly. Eight visible ventral plates, with straight anterior and posterior margins, becoming narrower posteriorly; strongly ventrally curved. Slight, scalloped ornament on posterior margin of tergites. No spinnerets or telson. Anal tubercle not resolved.

### (d) Cladistics

**Figure 3.**
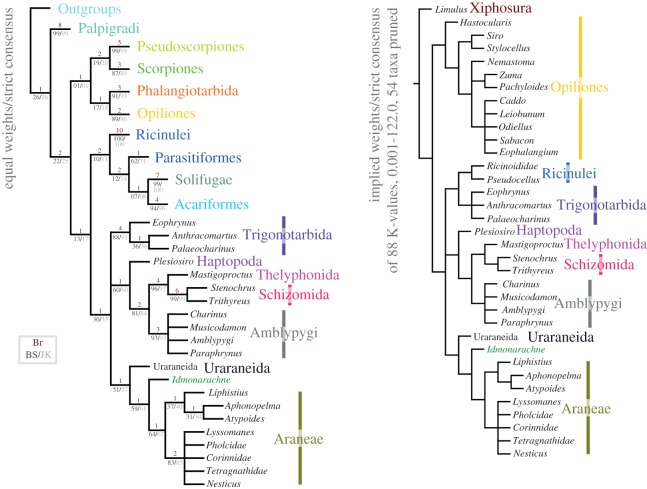
Cladistic analysis of the arachnids showing the position of *Idmonarachne brasieri* gen. et sp. nov. Both a strict consensus of the EW analysis, and a strict consensus of the trees recovered from a pruned dataset at 88 concavity constants, are presented, with the fossil in an identical position under all weighting schemes. See also the electronic supplementary material, SI figure S1.

The results of our cladistic analysis are presented in figure 3 and electronic supplementary material, SI figure 1. The EW analysis resulted in 256 most parsimonious trees of 463 steps. The strict consensus of these trees recovers *Idmonarachne brasieri* gen. et sp. nov. within the Pantetrapulmonata, as a sister group to the Araneae. The Uraraneida are recovered as a sister group to this clade. A Uraraneida + Araneae clade sister group relationship has previously been proposed [27] and named Serikodiastida [26]. Our *Idmonarachne* + Uraraneida + Araneae clade is defined by the presence of a pedicel, which is coded as present in this fossil based on a slight anterior tapering of the opisthosoma (figure 2a,*g*), and its position at a high angle to the prosoma, indicative of a weak prosoma–opisthosoma boundary. If this position resulted from the fossil being a moult, we would expect to see a suture splitting the dorsal from ventral prosoma. Coding the pedicel character as unknown results in both the Ururaneida and *Idmonarachne brasieri* gen. et sp. nov. resolving in a pantetrapulmonate polytomy with the spiders, trigonotarbids and Schizotarsata (Haptopoda plus Pedipalpi). The *Idmonarachne* + Uraraneida + Araneae clade is also defined by characters coded as unknown in the new taxon—the presence of a naked cheliceral fang, cheliceral venom gland, and opisthosomal silk glands and spigots. By inference based on this topology, all of these would have been present in *Idmonarachne*. The sister group relationship between *Idmonarachne* and Araneae is based on a long metatarsus (at least *ca* 1.5 times tarsus length), which is unique to these within the pantetrapulmonates. Details of the eyes in *Idmonarachne* are lacking, precluding strong support for its placement. However, jackknife and bootstrap values are higher for the *Idmonarachne* + Uraraneida clade, for example, than they are for an uncontroversial monophyletic clade for parasitiform mites. Furthermore, the *Idmonarachne* + Uraraneida + Araneae clade is consistent across a wide range of weighting parameters, being present in both equal weights analysis, and analyses at 88 concavity constants between 0.001 and 122.0 (consensus figure 3, four *K*-value trees shown in the electronic supplementary material, SI figure S1).

## 3. Discussion

**Figure 4.**
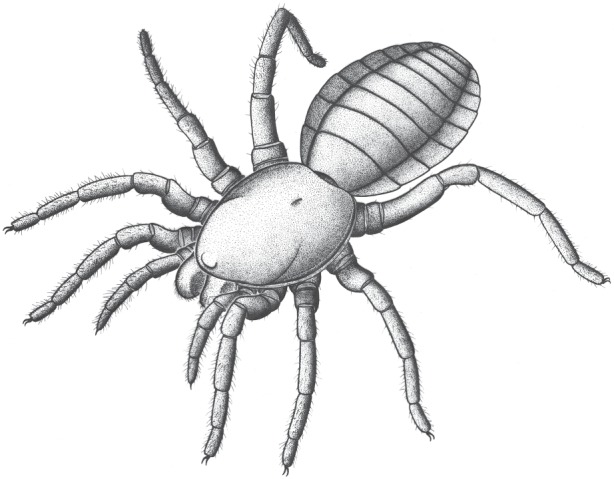
Suggested appearance of *Idmonarachne brasieri* gen. et sp. nov. in life.

*Idmonarachne* is clearly a pantetrapulmonate arachnid on the basis of its clasp-knife chelicerae and general habitus—an assertion supported by the cladistic analysis presented herein (idealized reconstruction shown in figure 4). The prosomal region is spider-like. This is seen in both the relatively large, forward-projecting chelicerae (figure 2*a*), with an oblique articulation, similar to the plagiognathic condition seen in mesothele spiders [17], and the nature of the prosomal appendages, as discussed in the morphological interpretation. However, the opisthosoma lacks evidence of spinnerets: a key autapomorphy of Araneae [2]. While this observation could be dismissed as taphonomic, the mesothele spider described from Montceau-les-Mines by Selden [12] revealed these structures quite clearly as holes in the matrix, which would be resolved with ease through tomographic investigation. No such holes are seen in the hand specimen of this fossil (figure 1), or revealed by the synchrotron scan which reveals the ventral surface in its entirety (figure 2*g*). Furthermore, the appendages are otherwise complete and well preserved. Hence we are confident that if opisthosomal projections were present in life, they would have been revealed in this study. Given that the new fossil is not a spider it must be a different species from the known Montceau-les-Mines mesothele *P. montceauensis* [12].

The cross section of the opisthosoma resembles that of a trigonotarbid. In this extinct arachnid order, the tergites are characteristically divided into median and lateral plates, and the latter often inflect upwards at the margins, yielding a similar profile to that of our new fossil. Elements of the anatomy are inconsistent with known trigonotarbids: (i) forward-projecting and quite large chelicerae visible from above and (ii) metatarsi longer than the tarsi. All of these factors are included in the updated matrix (electronic supplementary material, SI File 4), which unequivocally recovers *Idmonarachne* within Serikodiastida, as sister group to the spiders. This demonstrates that trilobate opisthosomal tergites have resulted from convergent evolution in at least three arachnid groups: the trigonotarbids, the ricinuleids and this taxon. Our cladistic analysis also allows reconstruction of character acquisition in the spider stem-group. The earliest-branching taxa resolved as stem-spiders herein are the uraraneids which possessed the ability to excrete silk, and an araneid habitus, including spider-like chelicerae. They also possessed a flagelliform telson. The latter was lost in *I. brasieri* (the most parsimonious assumption is that this species still possessed spigots), but the species retained plesiomorphic features such as tergites (also seen in mesothele spiders), and ventral plates (which are not). The Araneae then lost ventral plates, and evolved spinnerets with which they could spin silk: a key autapomorphy of the true spiders. Spinnerets significantly post-date the origin of silk, and multiple non-araneid groups with the ability to excrete silk existed. This innovation could be a key to the spiders' success—prior to this trigonotarbids, which went extinct during the Permian Period (299–252 Ma), appear to have greater species diversity [16]. Furthermore, this suggestion is reflected in the diversity of extant tetrapulmonate groups: there are 45 828 spider species [28], in contrast to 110 Thelyphonida, 161 Amblypygi and 260 Schizomida, which lack silk, but have broadly similar diversification times [29].

The morphology of *Idmonarachne* precludes placement within any known pantetrapulmonate group; it is an example of a Palaeozoic tetrapulmonate arachnid which does not fit comfortably into the established orders. This reflects the situation with the uraraneids, which were originally identified as trigonotarbids and then spiders, before being placed in their own order [2], and with problematic taxa like the Devonian *Xenarachne*, which is considered Tetrapulmonata *incertae sedis* [30]. The same is true of the extinct monotypic tetrapulmonate order Haptopoda, which is restricted to a handful of Carboniferous fossils [26]. Clearly, numerous extinct tetrapulmonate lineages existed during the Palaeozoic. This allows us to posit that the Late Carboniferous was a time with a greater diversity of pantetrapulmonate body plans, despite post-dating the origin of the Pantetrapulmonata by at least 100 Ma. Indeed, extinct orders such as the Uraraneida, Trigonotarbida and Haptopoda, coupled with taxa such as this and *Xenarachne*, suggest the period may have been a time of generally higher arachnid diversity than today.

Carboniferous species such as those of the family Arthromygalidae [31,32] are in need of restudy. Like *Idmonarachne*, these resemble spiders but apparently lack spinnerets. Hence the Carboniferous is a key time period to uncover stem-group spiders; future study focusing on these fossils will further inform our knowledge of critical periods in araneid evolution.

## 4. Material and methods

### (a) Material and photography

A single fossil from Montceau-les-Mines, MNHN.F.SOT110002 was photographed and scanned (Collection Sotty 2, deposited in the Muséum d'histoire naturelle d'Autun/Musée Jacques de la Comble, belonging to the Muséum National d'Histoire Naturelle, Paris). This is a partial void within a siderite nodule, split into two parts. In addition to siderite, an X-ray dense phase (likely pyrite) has formed spherical growths around parts of the fossil, and is also found as an irregular globular mass around the prosoma. White mineral infill is likely to be kaolinite [33]. The fossil was compared to Recent specimens of mesothele spiders (and other arachnids) held in the collections of the Museum für Naturkunde Berlin. The specimen was photographed with a Leica camera on a Leitz Aristophot and a fibre-optic light source, both dry and under 70% ethanol (to increase contrast), using Kodak Technical Pan (25 ASA) black and white film. Drawings were made using a Leica MZ12.5 stereomicroscope with a drawing attachment, and final illustrations were made in iDraw (www.indeeo.com).

### (b) Laboratory computed tomography

A CT scan was performed at the Natural History Museum (NHM), London on a Nikon HMX-ST 225. This employed a tungsten reflection target, a current/voltage of 145 µA/150 kV, no added filtration and 3142 projections at 0.7 s exposure. The resulting dataset has a 14.6 µm voxel size. A volume was created with CTPro v. 2.1.

### (c) Synchrotron computed tomography

To further investigate fine details, in particular the apparently soft, poorly resolved ventral surface of the opisthosoma, the specimen was scanned on Beamline I12 at Diamond Light Source, UK [34]. We note that this is the first time that synchrotron radiation investigation of a siderite-hosted fossil has been reported. The fossil was imaged using 0.124 Å (100 keV) X-rays, and a custom-built X-ray camera including an X-ray-sensitive scintillator emitting visible light (cadmium tungstate), visible light optics and a PCO4000 camera, with scientific grade 4008 × 2672 pixel CCD sensor.

A series of 1800 projection images were collected at 0.1° intervals through a 360° rotation. The camera was placed 2000 mm beyond the sample, to record differential X-ray phase contrast [35]. The phase image was retrieved using the method of Paganin *et al*. [36] to improve contrast. Ring artefacts caused by scintillator defects were removed through the combination of projections at 180° rotation to each other. The reconstructed three-dimensional volumes based on the filtered back-projection algorithm were implemented by in-house Mathematica codes [37] and the filter of Raven [38] applied to further clean small ring artefacts. The selected microscope optics of the beamline provided a voxel size of 5.0 µm.

### (d) Reconstruction

Digital visualizations of the tomographic datasets were created using the SPIERS software suite [39] following the methods of Garwood & Sutton [40]; additionally, in the laboratory-scan the opisthosoma was manually traced as the thin cuticle could not be thresholded, and then rendered as partially transparent in the finished model. The reconstruction from the laboratory-scan includes the fossil in its entirety, whereas only anatomical features of interest have been reconstructed from the synchrotron data. Isosurfaces were ray-traced in Blender [41]. Reconstructions are presented in the interchange format VAXML [39] as electronic supplementary material, SI File 01 (via Dryad, http://dx.doi.org/10.5061/dryad.v089t/1). A rendered animation is presented as electronic supplementary material, SI File 02 (via Dryad, http://dx.doi.org/10.5061/dryad.v089t/2).

### (e) Cladistic analysis

The fossil was coded into a modified version of the dataset of Garwood & Dunlop [26], to assess its affinities (character statements in the electronic supplementary material, SI File 03, full matrix SI File 04).

Analyses were performed in the software package TNT v.1.1. ([42]; made available with the sponsorship of the Willi Hennig Society). This employed a traditional search: tree bisection–reconnection (TBR) with 1000 replicates, saving 100 trees per cycle. Multistate characters were unordered. We present strict consensus trees with both equal weighting (EW) and implied weights (IW) [43]. For the latter, to test the stability of the fossil's placement, we present a strict consensus of trees recovered at 88 concavity constants (spanning *k* = 0.001 to *k* = 122.0). When all taxa are included, most arachnid orders are recovered as monophyletic, but the relationships between them a polytomy. The placement of the fossil is identical to the EW analysis. Hence for clarity we present a strict consensus tree of a matrix pruned to include just pantetrapulmonates and Opiliones, with *Limulus* as an outgroup (Matrix: electronic supplementary material, SI File 05). The fossil's position is identical in the strict consensus under all tested analytical parameters. For the EW analyses, TNT was used to calculate jackknife ([44]; 33% removal probability, 1000 replicates), and bootstrap ([45]; 1000 replicates) support values with nodal support given as absolute frequencies. Bremer support values [46] were also generated in TNT using the inbuilt Bremer supports analysis (absolute supports, TBR from existing trees saving up to 10 steps suboptimal).

## Supplementary Material

Garwood_et_al_SI_File_03_Character_list_resub.pdf

## Supplementary Material

Garwood_et_al_SI_File_04_Full_matrix_resub.txt

## Supplementary Material

Garwood_et_al_SI_File_05_Pruned_matrix_resub.txt

## Data accessibility

The datasets supporting this article have been uploaded as part of the electronic supplementary material: SI File 01 (available via Dryad, http://dx.doi.org/10.5061/dryad.v089t/1), using the VAXML interchange format [39]. Matrices—as well as being included in SI—are included in Morphobank (Project 2375).
